# Autofluorescence-based sorting removes senescent cells from mesenchymal stromal cell cultures

**DOI:** 10.1038/s41598-020-76202-2

**Published:** 2020-11-05

**Authors:** Alessandro Bertolo, Julien Guerrero, Jivko Stoyanov

**Affiliations:** 1 Swiss Paraplegic Research, 6207 Nottwil, Switzerland; 2 Tissue Engineering for Orthopaedics and Mechanobiology (TOM), Department for Biomedical Research (DBMR), University of Bern, 3008 Bern, Switzerland

**Keywords:** Biological techniques, Cell biology, Molecular biology, Stem cells, Biomarkers, Medical research, Molecular medicine

## Abstract

Mesenchymal stromal cells (MSC) are used in cell therapy, but results depend on the unknown quality of cell populations. Extended culture time of MSC increases their senescent levels, leading to a critical loss of cell fitness. Here, we tested the suitability of MSC-sorting based on their FACS autofluorescence profile, for a rapid and non-invasive method of senescent cell elimination. Cells were classified in low- (LA) and high- (HA) autofluorescence groups, and results compared to the original MSC population (control). Three days after sorting, cells were screened by replicative senescence markers (cell volume, SA-β-Gal assay and gene/protein expression) and MSC differentiation assays. The transcriptional profiles of sorted MSC were also analyzed by RNA‐Seq. Compared to control, LA cells had 10% lower cell volume and autofluorescence, and 50% less SA-β-Gal + cells. Instead, HA cells had 20% higher cell volume and autofluorescence, and 120% more SA-β-Gal + cells. No changes in replicative senescence and differentiation potentials were observed between all groups. However, 68 genes (16 related to senescence) were significantly differentially expressed (DEG) between LA and other groups. Biological network of DEG identified CXCL12 as topological bottleneck. In summary, MSC sorting may have practical clinical implications to enhance the results of MSC-based therapies.

## Introduction

Human mesenchymal stromal cells (MSC) are adult cells related to the musculoskeletal lineage, which in the recent years were associated numerous times to future therapeutical application, thanks to their fast replicative rate^1^, differentiation potential into mesodermal lineages^2^ and secretion of trophic and anti-inflammatory factors^3^. The isolation of MSC—according to current criteria^4^—produces heterogeneous, non-clonal cultures of stromal cells containing stem cells with different multipotential properties, committed progenitors and differentiated cells^5^. Moreover, the use of MSC in regenerative therapies is hampered by the low starting number of MSC that can be isolated from adult tissues. The initial pool and functionality of MSC could be further reduced in quantity and quality (stemness) by diseases such as osteoporosis^6^, leukaemia^7^, or lung and breast cancers^8^. Among the factors underlying the loss of functionality of MSC in tissues, aging must also be seen as a major factor adversely affecting the quality of cells in tissues^9,10^. Nonetheless, decreased tissue regeneration is associated with an increased number of senescent cells, eventually having a negative impact on life expectancy^11^.

The mechanism leading to senescent phenotypes is triggered by multiple events, such as oxidative stress, DNA damage, telomere shortening, and oncogene activation^12,13^. It protects the dividing cells from undertaking a tumorigenic transformation^14^. MSC proliferative capacity is reduced and MSC in a senescent state secrete pro-inflammatory factors and facilitate the invasion of cancer cells into tissues^15^. Similarly to in vivo ageing, a significant proportion of MSC in vitro will also grow into a senescent phenotype when cultured for prolonged period of time^16^. As a result, the effectiveness and reliability of MSC in tissue regeneration may decrease due to age-related changes, both in vivo and in vitro, leading to a potential reduction in the MSC useful for cell-based therapy. This is relevant, in particular, for the medical use of autologous stem cell transplantation in elderly patients.

For efficacious therapies, a large number of cells are required, resulting from an extensive ex vivo cell expansion, and possibly leading to the accumulation of several senescent MSC phenotypes in culture. Identifying a tool for detecting and removing senescent cells in vitro would therefore be necessary for the development of new and more efficient cell-based therapies. Cell autofluorescence is optimal biomarker for screening and targeting senescent in MSC as it does not require additional cell staining. Our research has shown that MSC autofluorescence is directly related to cellular senescence measurements^17^.

Based on the above information, in this study we have sorted MSC based on their autofluorescence in order to obtain a cell populations either cleared of- or enriched in- senescent cells, to test the idea of using autofluorescence for targeting cellular senescence. This process has a potential as a clinically relevant method for removing senescent cells and isolating functionally robust primary MSC subpopulation.

After sorting, MSC culture was divided into three groups characterized by low (LA), mid (MA) and high (HA) autofluorescence, and the findings were compared to unsorted control populations (processed or unprocessed by FACS). Cellular senescence was assessed by cell volume, SA-β-Gal assay with chromogenic substrate (X-GAL)^18^, gene and protein expression of cell cycle regulator markers associated to cell senescence (p16^INK4A^, p18^INK4C^, p21^CIP1^, p53, Rb, E2F1, ANKRD1 and CDCA7)^19–22^. Also variations in MSC differentiation potential were investigated by adipogenic, chondrogenic and osteogenic differentiation assays^23^. Finally, using RNA sequencing (RNA-Seq), we analysed four MSC groups (control, unsorted, LA and HA) isolated from a single donor in order to identify all differences in gene expression after autofluorescence sorting.

## Results

### Characterization of autofluorescence-based sorting of mesenchymal stromal cells (MSC) groups

**Figure 1:**
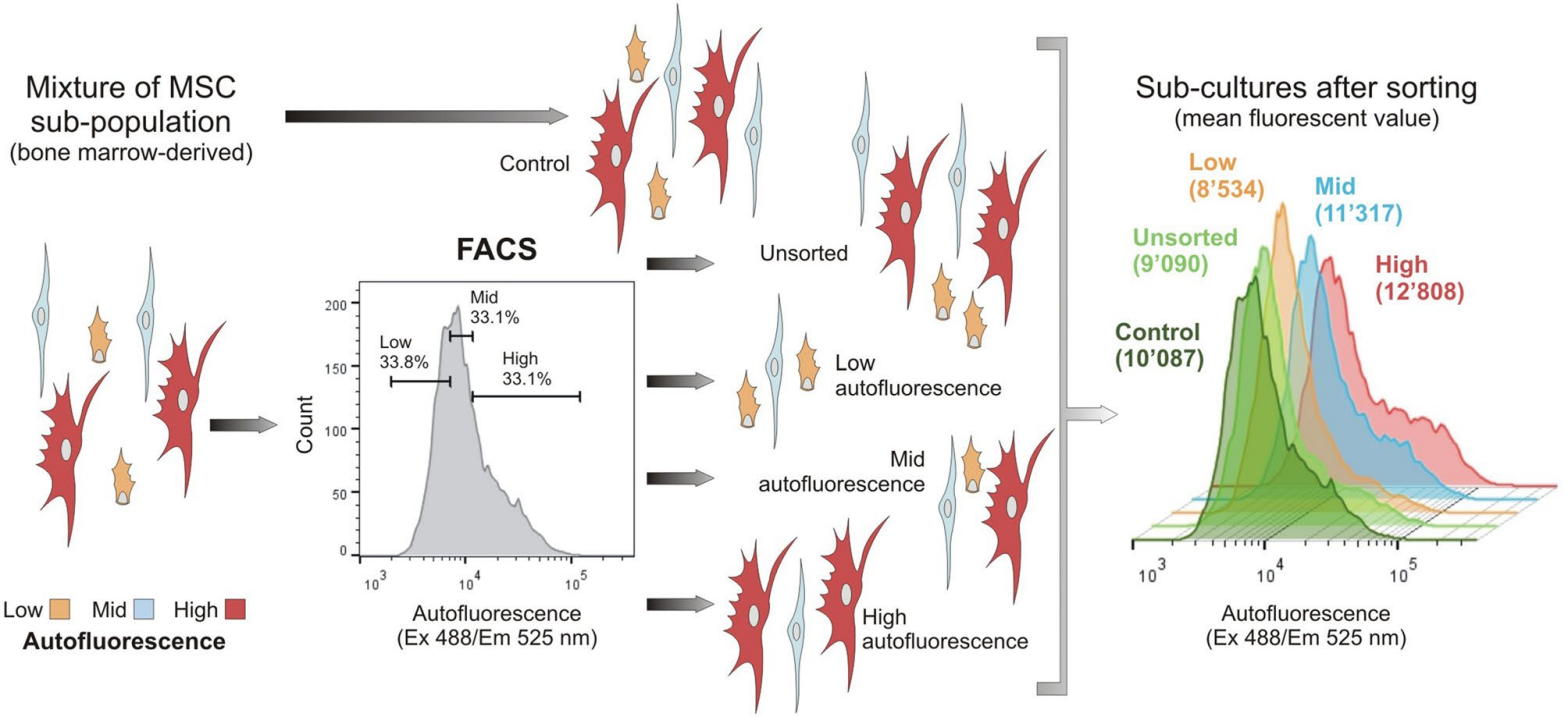
Schematic representation of the protocol for sorting MSC based on autofluorescence. Cellular autofluorescence is used to discriminate between cell types, with smaller cells having the lowest autofluorescence in comparison to the largest ones. Based on this assumption, we divided bone marrow derived MSC (n = 3) using a FACS in three equal groups (~ 1/3 each): LA (in yellow), MA (in blue) and HA (in red) groups. As controls, the original MSC population was either propagated in culture (control) or passed through the FACS, but without sorting (unsorted). After FACS, the cells were collected, expanded in culture and analysed after 3 days. Diagram created with PowerPoint 2016 (Microsoft). (Abbreviations: MSC = mesenchymal stromal cells; FACS = fluorescence-activated cell sorting; LA = low autofluorescence; MA = mid autofluorescence; HA = high autofluorescence).

**Figure 2:**
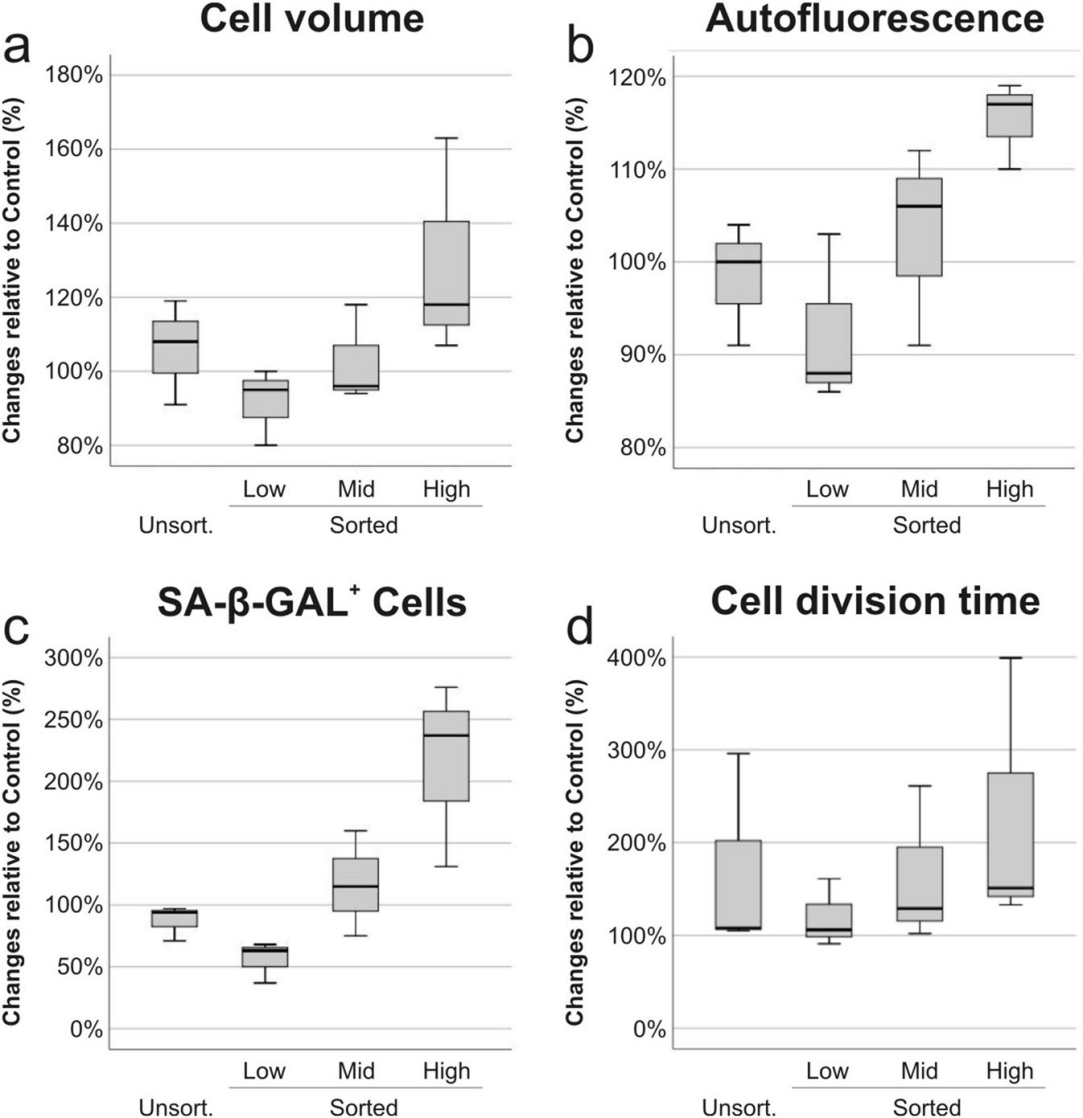
Phenotypic characterization of MSC senescence. The phenotypic analysis of MSC in unsorted and sorted groups—low (LA), mid (MA) and high (HA) autofluorescence—was conducted 3 days after sorting by comparing to control cell volume (**a**), cellular autofluorescence (**b**), SA-β-Gal activity (**c**) and cell division time (**d**).

**Figure 3:**
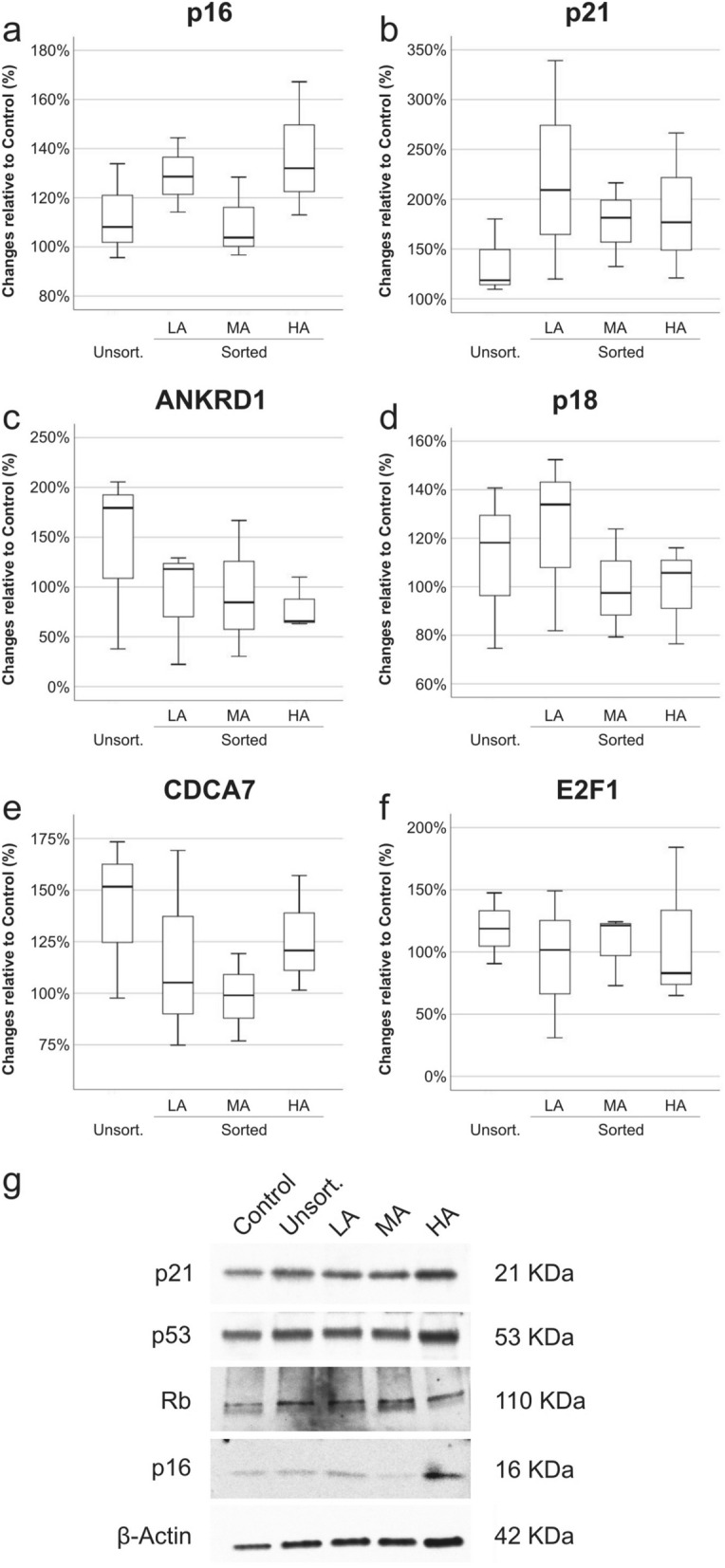
RNA and protein expression of MSC-related senescence markers. The expression of senescence markers in MSC (n = 3) was analysed in unsorted and sorted groups—LA, MA and HA—and compared to control. Gene expression of positive—p16^INK4A^ (**a**), p21^CIP1^ (**b**) and ANKRD1 (**c**)—and negative— p18^INK4C^ (**d**) CDCA7 (**e**) and E2F1 (**f**)—senescence markers was tested by qPCR (normalized to beta-actin and PPIA). The protein expression of p21^CIP1^, p53, Retinoblastoma (Rb) and p16^INK4A^ was compared between control, unsorted and sorted MSC groups by semi-quantitative Western blot (**g**).

**Figure 4:**
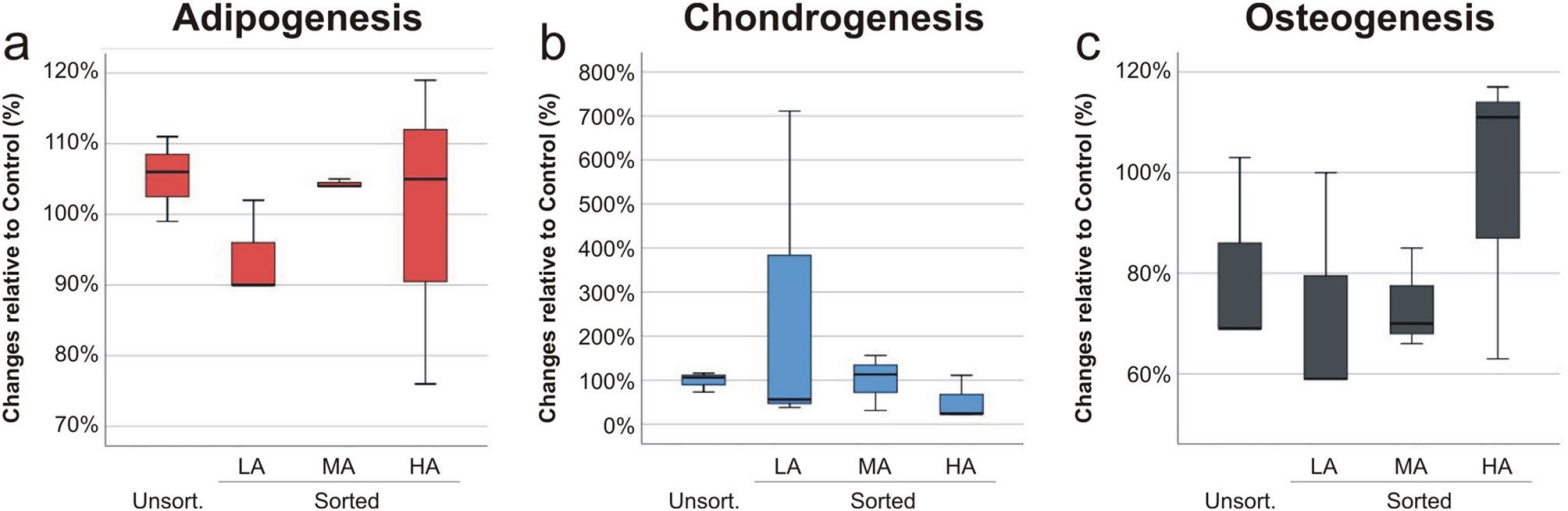
Differentiation potential characterization of MSC. The differentiation potential of MSC (n = 3) to adipogenic (**a**), chondrogenic (**b**) and osteogenic (**c**) lineages was evaluated after 21 days of culture by quantitative staining of fat vacuole formation (Oil red O), accumulation of proteoglycan (alcian blue staining) and calcium accumulation (von Kossa staining) respectively.

Bone marrow isolated MSC (n = 3) were initially expanded to obtain a large number of cells to perform the sorting via FACS. During the sorting, cells were separated in three groups: low- (LA), mid- (MA) and high (HA) autofluorescence (Fig. 1). As comparison, the original MSC population was propagated in culture with (group unsorted) or without (group control) passing through the FACS. In order to characterize the five MSC groups, we investigated markers of phenotypic (Fig. 2) and genetic senescence (Fig. 3), and differentiation potential (Fig. 4), after three days in culture following cell sorting.

Senescent MSC show clear morphological features, such as enlarged aspect^24^ and cell volume (Fig. 2a), higher cellular autofluorescence^17^ (Fig. 2b), enhanced senescence-associated beta-galactosidase (SA-β-Gal) activity (Fig. 2c) and longer cell division time (Fig. 2d). In comparison to the control group, average cell volume and autofluorescence were reduced in LA cells (10%), and increased in HA cells (30% and 20% respectively). The average proportion of SA-β-Gal positive cells, as a percent of the total population, was remarkably decreased in LA group (~ 50%) and increased in HA group (~ 10%). In the unsorted and MA groups, the average variations in cell volume, autofluorescence and SA-β-Gal positive cells were always in the ± 10% range compared to control. Only cell division time was longer for all groups compared to control, which may be due to the cell sorting process. In LA group the time increase was ~ 20%, in the unsorted and MA groups ~ 70% and in the HA group was ~ 130%. However, in the following culture passages, we observed a gradual convergence of all groups to control, eventually all groups showing the same senescent profile (data not shown).

To further define MSC features, we evaluated the gene expression of markers classically associated to replicative senescence (Fig. 3a–f). Gene expression levels correlated positively (p16^INK4A^, p21^CIP1^ and ANKRD1) and negatively (p18^INK4C^, CDCA7 and E2F1) with MSC replicative senescence. In all groups, we observed small-to-moderate average increases in p16^INK4A^ (range, 10–40%) and ANKRD1 (0–40%) as compared to control. On the other hand, the average expression of p21^CIP1^ was nearly doubled in all groups, ranging from ~ 40% increase in the unsorted group to over 120% increase in the LA group. Also minor changes in the expression of p18^INK4C^, CDCA7 and E2F1 were found. Compared to control, we observed an increased average expression of p18^INK4C^ (0–20%), CDCA7 (0–40%) and E2F1 (0–20%) in all groups. Protein extracts from all MSC groups were used to assess changes in expression of p21^CIP1^, p53, retinoblastoma (Rb) and p16^INK4A^ by western blot (Fig. 3g). Quantification of bands—normalized to β-actin—showed that in comparison to control, MSC in HA group had higher expression of p16^INK4A^ (4.5-fold), and lower expression of Rb (-1.0-fold). No relevant changes (± onefold) were observed in the other groups, except the lower expression of p16^INK4A^ in the MA group (-1.3-fold).

We also investigated the ability of MSC to differentiate towards adipogenic (Fig. 4a), chondrogenic (Fig. 4b) and osteogenic (Fig. 4c) lineages after 21 days in culture. In comparison to control, we observed negligible changes in MSC adipogenic differentiation in all groups, with average variations of ± 5%, assessed and quantified by Oil red O staining. Analysis of chondrogenic potential, evaluated by accumulation of proteoglycan and quantified by alcian blue staining, showed no changes in the unsorted and MA groups compared to control, but an average decline of ~ 50% in the HA group and an increase of ~ 250% in the LA group (despite high variation within the data). Osteogenic potential of MSC was evaluated by measuring calcium deposition within the extracellular matrix and, in comparison to control, all groups were in average 25% less potent, except HA group which equalled the control.

### RNA sequencing (RNA-Seq) of sorted and unsorted MSC

To investigate differences in the gene transcription profile of MSC after sorting, we performed genome-wide RNA-seq analysis. RNA was isolated from sorted (LA and HA groups) and unsorted (control and unsorted) MSC, after three days of culture following FACS. Data from RNA-seq were plotted in multiple methods, such as dispersion (Supplementary Fig. S1a), principal component analysis (PCA) and multi-dimensional scaling (MDS), to evaluate the quality of RNA-seq output, as well as the relationships between sorted and unsorted MSC (Supplementary Fig. S1b).

**Figure 5:**
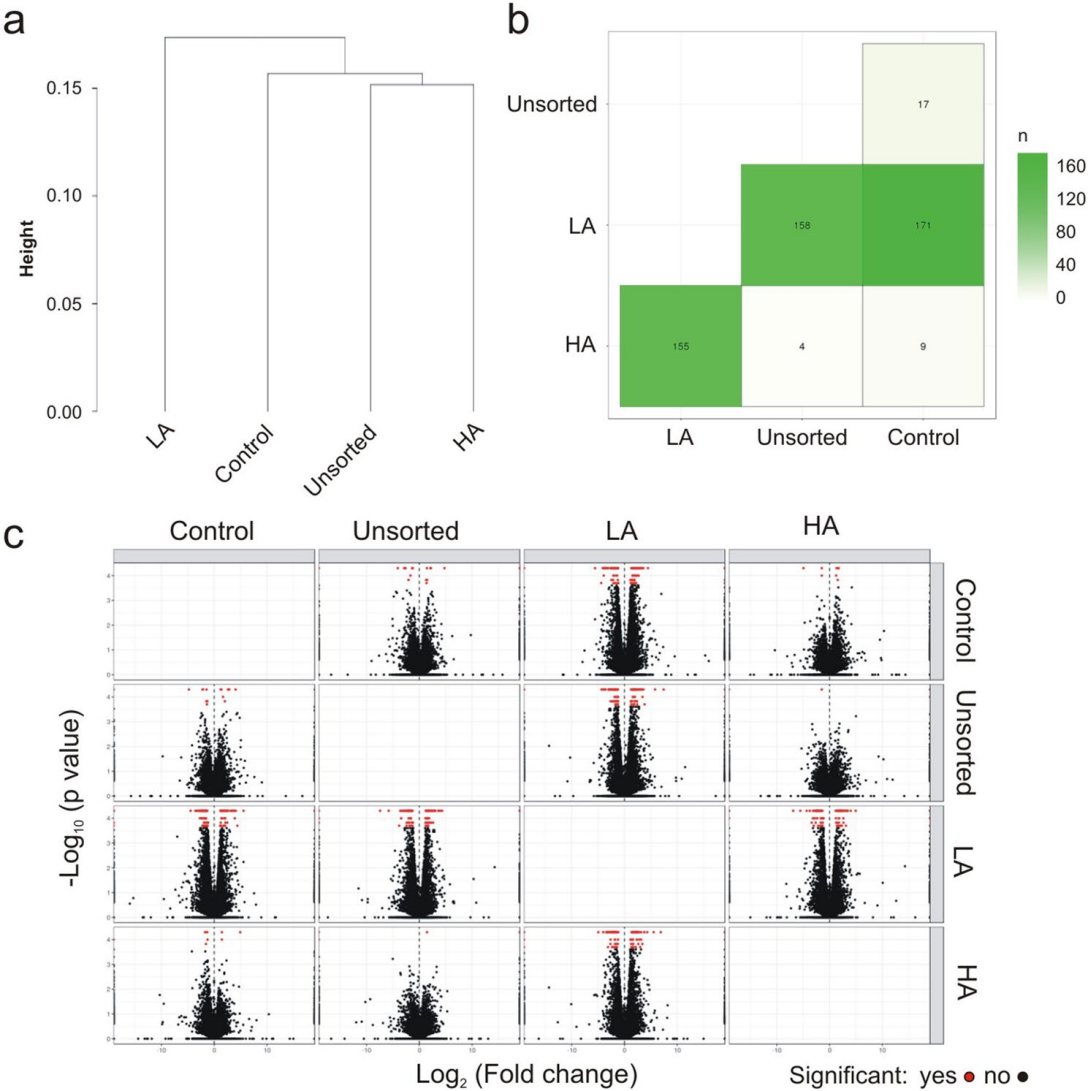
Identification of differentially expressed genes (DEG) between MSC groups. The hierarchical clustering analysis of MSC transcriptomes obtained using RNA-seq data is shown in (**a**). The exact number of DEG among groups is summarized in a table (**b**) and volcano plots (**c**) report the relationship between fold-changes and significance levels. Each dot represents a DEG, in red significant and in black non-significant.

**Figure 6:**
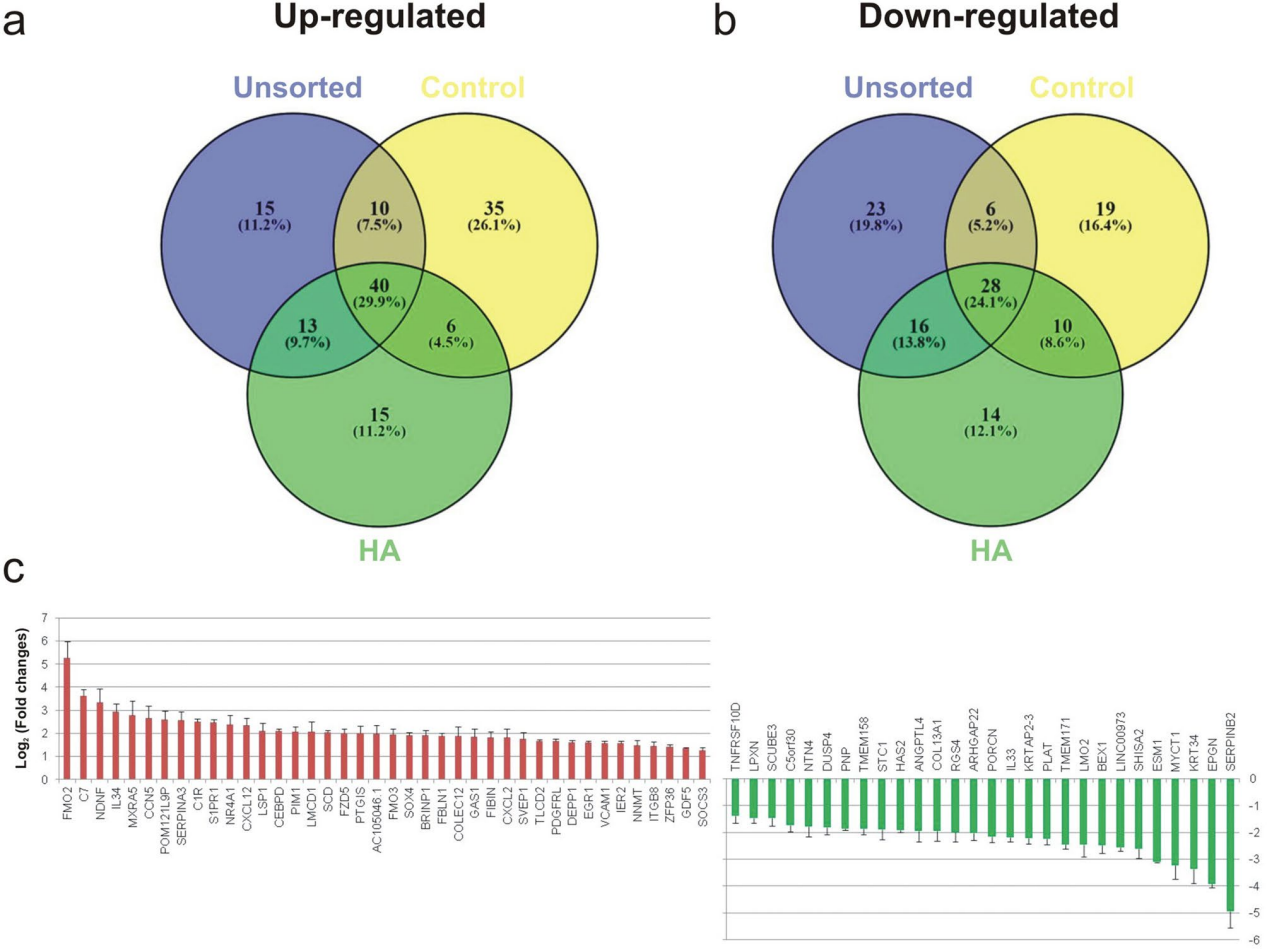
DEG shared from unsorted, control and high autofluorescence (HA) groups, compared to low autofluorescence (LA) group. Comparison of DEG between groups showed that 68 DEG were in common among all groups, as represented by the Venn diagram (the number of DEG is indicated in the diagram). Diagram created with PowerPoint 2016 (Microsoft). 40 DEG were up-regulated (**a**), while 28 were down-regulated (**b**), and then sorted according to fold changes (**c**).

We identified differentially expressed genes (DEG) between all groups using Cuffdiff program (*p* value < 0.0005; |log_2_|fold change > 1), and results are represented schematically by dendrogram of hierarchical clusters (Fig. 5a), table (Fig. 5b), and volcano plots (Fig. 5c). RNA-seq data showed that HA and unsorted cells were similar and closer to control, while LA cells were distinct from other groups. We observed only 9 and 17 genes differentially expressed in HA and unsorted groups respectively, compared to control. These results indicated the absence of discernible phenotypes between control and unsorted groups, demonstrating the lack of negative influences of FACS processing on MSC. In contrast, 171 genes in the control group were significantly changing in expression, compared to LA group. Always in comparison to LA cells, 158 and 155 genes were differentially expressed between unsorted and HA groups respectively. Among these genes, we observed 40 upregulated (Fig. 6a) and 28 downregulated (Fig. 6b) genes that were shared among all three groups (Fig. 6c). Of the 68 DEG, 16 genes (ITGB8, COL13A1, DUSP4, MYCT1, ESM1, FMO2, FMO3, NDNF, C1R, ESM1, CXCL12, VCAM1, NTN4, PLAT, KRT34, SERPINB2) have been already associate to senescence in MSC in vitro^25–28^ and in vivo^29,30^.

### Identification of DEG among LA and HA cells using RNA‑Seq

**Figure 7:**
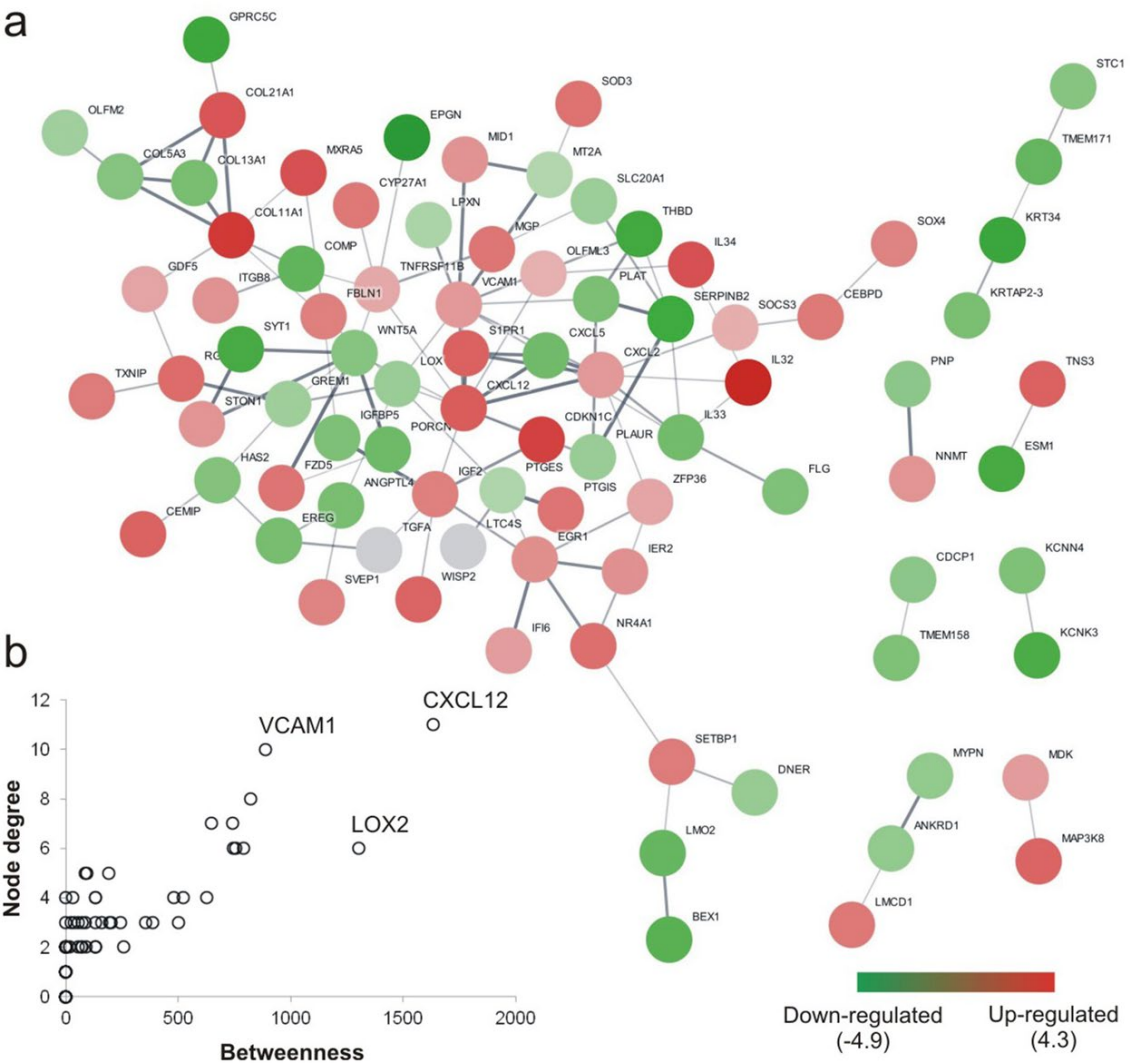
Network of DEG between low (LA) compared to high (HA) autofluorescence groups. A total of 155 DEG were screened in MSC from LA and HA groups and protein/protein interactions were identified and generated by the STRING database (**a**). Gradual shift of colour from green to red indicates expression values change from low to high. Identification of bottlenecks in the network was determined by comparing node degree and betweenness (**b**).

Based on system biology analysis, we identified the interaction network formed by the 155 DEG from the comparison between LA and HA cells (Fig. 7a) and the genes CXCL12, VCAM1 and LOX2 were recognized as a bottlenecks (Fig. 7b). The 40 genes with the greatest fold changes and significant *p* value are shown in Table 1.

**Table 1:** Twenty genes with the greatest fold increase (left columns) and decrease (right columns) in the LA group in comparison to HA group.

20 genes with greatest fold increase				20 genes with greatest fold decrease			
Gene name	Fold change (Log_2_)	*p* value	FDR	Gene name	Fold change (Log_2_)	*p* value	FDR
B3GNT8	6.89	5.00E–05	0.0151	SERPINB2	− 4.95	5.00E–05	0.0151
FMO2	5.54	5.00E–05	0.0151	EPGN	− 4.01	5.00E− 05	0.0151
IL32	4.33	5.00E− 05	0.0151	PODXL	− 3.45	5.00E− 05	0.0151
NDNF	3.75	5.00E− 05	0.0151	GPRC5C	− 3.37	5.00E− 05	0.0151
C7	3.74	5.00E− 05	0.0151	KRT34	− 3.35	5.00E− 05	0.0151
MIR646HG	3.39	1.50E− 04	0.0359	AC097451.1	− 3.28	5.00E− 05	0.0151
COL11A1	3.16	1.00E− 04	0.0267	THBD	− 3.24	5.00E− 05	0.0151
AC022784.1	3.14	5.00E− 05	0.0151	MYCT1	− 3.15	5.00E− 05	0.0151
CDKN1C	2.99	2.00E− 04	0.0436	ESM1	− 3.12	5.00E− 05	0.0151
TPD52L1	2.94	5.00E− 05	0.0151	SYT1	− 3.11	2.00E− 04	0.0436
SERPINA3	2.85	1.00E− 04	0.0267	KCNK3	− 3.04	5.00E− 05	0.0151
MXRA5	2.71	5.00E− 05	0.0151	CCDC178	− 2.95	5.00E− 05	0.0151
IL34	2.70	5.00E− 05	0.0151	BEX1	− 2.78	5.00E− 05	0.0151
C1R	2.64	5.00E− 05	0.0151	SHISA2	− 2.70	5.00E− 05	0.0151
COL21A1	2.63	1.50E− 04	0.0359	LNCOG	− 2.66	1.50E− 04	0.0359
POM121L9P	2.57	5.00E− 05	0.0151	COMP	− 2.63	1.00E− 04	0.0267
CXCL12	2.51	5.00E− 05	0.0151	TMEM171	− 2.58	5.00E− 05	0.0151
LSP1	2.45	5.00E− 05	0.0151	LMO2	− 2.55	5.00E− 05	0.0151
TNS3	2.43	5.00E− 05	0.0151	INA	− 2.47	5.00E− 05	0.0151
S1PR1	2.43	5.00E− 05	0.0151	LINC00973	− 2.40	5.00E− 05	0.0151

*FDR* false discovery rate.

**Figure 8:**
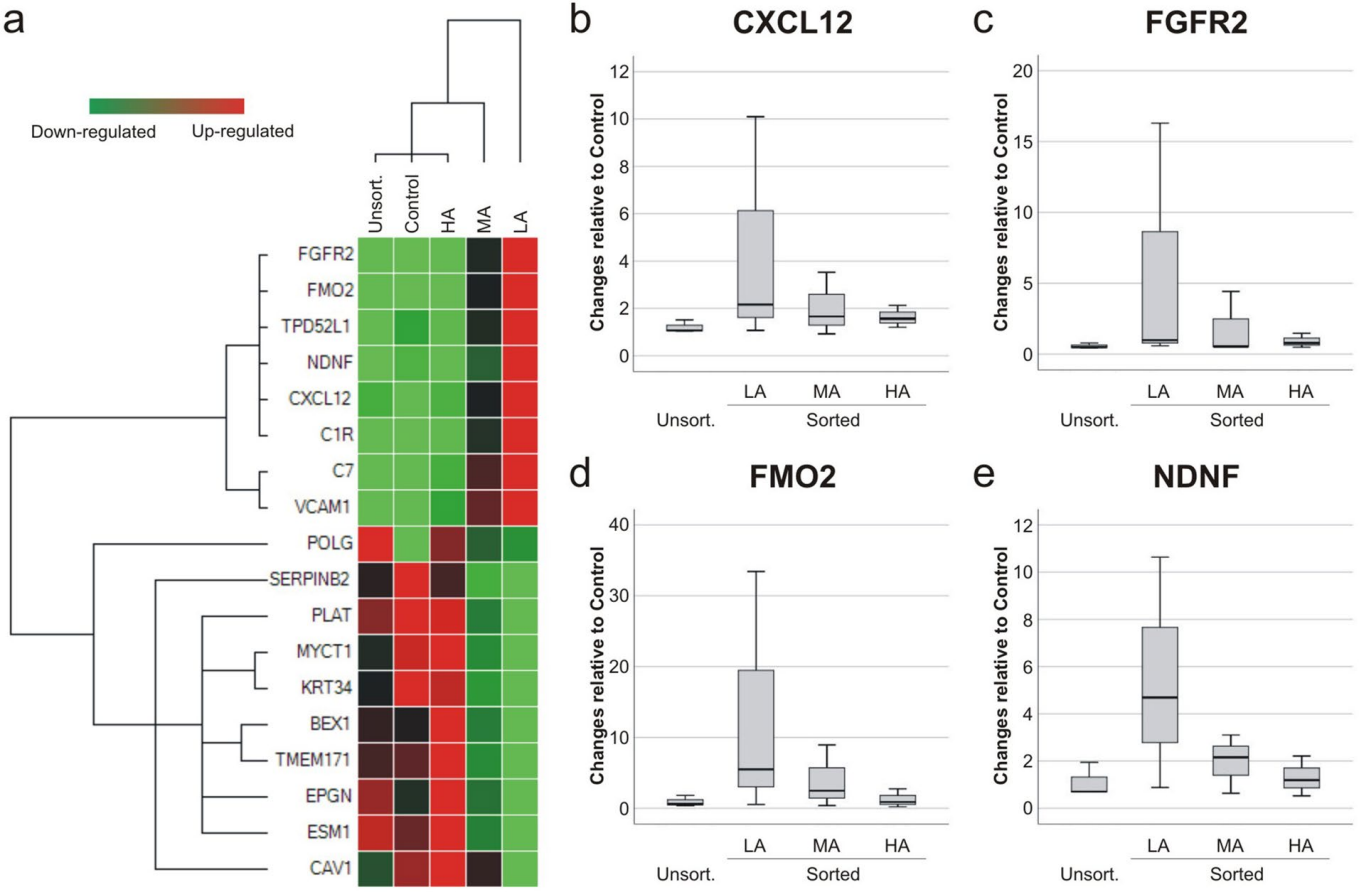
Validation by qRT-PCR of DEG identified by RNA-Seq. Hierarchical clustering analysis (**a**) of 18 genes with the highest expression changes between low (LA) and high (HA) autofluorescence groups (n = 1). Analysis included all groups: control, unsorted, LA, HA, and mid autofluorescence (MA). Gradual shift of colour from green to red indicates expression values change from low to high. The expression of senescence markers CXCL12 (**b**), FGFR2 (**c**), FMO2 (**d**) and NDNF (**e**) was further validated (n = 3). Gene expression results were compared to control group and normalized to beta-actin and PPIA.

The 155 differently expressed genes were analysed by ToppGene and were found to be involved in a number of biological processes, such as blood vessel development and cellular response to cytokine stimulus (Table 2). Gene ontology (GO) term analysis of the DEG revealed that the most significant associations were with extracellular matrix and cell receptor regulatory activity. Overall, the results showed that the differences between groups reside in the way cells communicate and react with the environment, which is the peculiarity of MSC paracrine action. To validate RNA-Seq results, we evaluated by qRT-PCR the gene expression of the 18 genes with greatest fold changes between LA and HA cells, and represented in the clustergram (Fig. 8a). The validation analysis confirmed the results of the RNA-seq, and the hierarchical clustering analysis of the data also established LA group as distinct to the control, unsorted and HA groups, with MA group standing in the middle. Genes FGFR2, FMO2, TDPD52L1, NDNF, CXCL12, C1R, C7, VCAM1 were upregulated in the LA group, whereas POLG, SERIPNB2, PLAT, MYCT1, KRT34, BEX1, TMEM171, EPGN, ESM1, CAV1 were upregulated in the HA group.

**Table 2:** Gene Ontology (GO) categories with the lowest *p* values associated to DEG between LA and HA groups.

	Name	ID	*p* value	FDR	Hit count
**GO: biological process**					
1	Blood vessel development	GO:0001568	4.40E−09	8.60E−06	24/849
2	Cellular response to cytokine stimulus	GO:0001944	1.04E−08	8.60E−06	28/1188
3	Skeletal system development	GO:0071345	2.25E−08	1.14E−05	19/582
4	Positive regulation of cell population proliferation	GO:0048514	3.35E−08	1.32E−05	26/1096
5	Negative regulation of response to external stimulus	GO:0072358	6.68E−08	1.97E−05	16/437
**GO: cellular component**					
1	Extracellular matrix	GO:0031012	3.83E−14	1.11E−11	26/59
2	Collagen-containing extracellular matrix	GO:0062023	1.20E−12	1.73E−10	22/47
3	Clathrin-coated vesicle membrane	GO:0030665	1.54E−05	1.49E−03	7/11
4	Collagen trimer	GO:0005581	2.86E−05	2.06E−03	6/8
5	Cell surface	GO:0009986	2.37E−04	1.34E−02	18/103
**GO: molecular function**					
1	Receptor regulator activity	GO:0030545	6.81E−08	1.07E−05	18/547
2	Sulphur compound binding	GO:1901681	7.58E−07	8.81E−05	12/279
3	Integrin binding	GO:0005178	1.02E−06	8.81E−05	9/146
4	Growth factor activity	GO:0008083	3.12E−06	2.06E−04	9/167
5	N,N-dimethylaniline monooxygenase activity	GO:0004499	3.49E−06	2.06E−04	3/5

*FDR* false discovery rate.

Next, we investigated in all three MSC donors the expression of the genes CXCL12^26^ (Fig. 8b), FGFR2^28^ (Fig. 8c), FMO2^31^ (Fig. 8d) and NDNF^32^ (Fig. 8e) which had the largest fold increase in LA and HA cells and which have been shown to be important MSC senescence-related genes. In comparison to control, the average expression of CXCL12 (4.4 fold), FGFR2 (5.9 fold), FMO2 (13.6 fold) and NDNF (5.4 fold) was the highest in the LA group. In the MA group the average upregulation of CXCL12, FGFR2, NDNF (all ~ 2.0 fold) and FMO2 (3.9 fold) was less consistent, while in the HA and unsorted groups gene expression changes were minimal (± 0.3 fold).

## Discussion

In this study, we showed that endogenous autofluorescence of human mesenchymal stromal cells (MSC) could be used as a non-invasive and easily applicable method to remove senescent cells from in vitro cultures. As we have previously demonstrated^17^, when cells become senescent in vitro they significantly change their phenotype and they accumulate undegredable molecules and key endogenous fluorophores—such as free nicotinamide adenine dinucleotide (NADH), flavin adenine dinucleotide (FAD) and lipofuscin^33^—resulting in a progressive increase of cellular autofluorescence.

Native cell fluorescence consists of a mixture of molecules that naturally display fluorescence, resulting in detailed fluorescent finger prints. For instance, by bioimaging of the autofluorescence of murine retinal cells—without any external biomarkers—one can identify biochemical constituents, their location and abundance within cytoplasm^34^. Likewise, based on this distinct autofluorescent signature, in our study we provide evidences that MSC with low average autofluorescence (LA) were less senescent than cell with higher autofluorescence (HA). In comparison to HA cells, LA cells were morphologically smaller, halved the proportion of senescence-associated-β galactosidase (SA-β-gal) positive cells and overexpressed genes negatively associated with cellular senescence, such as CXCL12, FGFR2, FMO2 and NDNF^26,28,31,32^. However, HA cells did not exhibit the entire phenotype usually associated with replicative senescence. The gene expression of the characteristic markers of replicative senescence—positive genes p16^INK4A^, p21^CIP1^ and ANKRD1 and negative genes p18^INK4C^, CDCA7 and E2F1—were unaffected in comparison to control. Only in the HA group compared to other groups the protein expression of p16^INK4A^ and Rb was higher and lower respectively. This means, that the autofluorescence method selects senescent cells based on a different phenotype, which do not involve substantial differences in cell cycle transcriptional regulators.

Indeed, RNA‐Seq transcriptome of HA cells found overexpression of genes considered senescent markers, but with no known function related to replicative senescence, such as Plasminogen Activator, tissue type (PLAT)^28^, or indirect function, such as serine protease inhibitor-B2 (SERPINB2) that binds to p21^CIP1^ preventing its proteasome-mediated degradation^25^. Such more granulated level of evidence suggests that this senescence was either induced by different stimuli or detected before the classical cell cycle arrest/progression occurs. Indeed, comparing LA and HA cells, we see significant changes in cellular response to cytokine stimulation and receptor regulatory activity, as well as remodeling of extracellular collagen (Table 2), indicating a more likely presence of cell paracrine senescence caused by senescence-associated secretory phenotype (SASP). Previously, we demonstrated the significant increased secretion of IL-6 and MCP-1—involved in the SASP^35^—in those MSC with the highest cellular autofluorescence^17^. As a matter of fact, we have observed that after several weeks in culture, MSC in all groups aligned and converged to similar senescent levels to these in the control, despite significant differences in senescent frequencies few days after sorting. We suppose that autofluorescence-based sorting of cells interfered with the equilibrium and ratio between sub-populations of MSC, and as a compensatory mechanism, the secretion of SASP factors influenced the behavior of neighboring cells in a paracrine fashion, especially in the HA group.

Among SASP factors, we found that CXCL12 (aka SDF-1; stromal cell-derived factor-1) was the bottleneck (the central node) in the network of differentially expressed genes between LA and HA cells (Fig. 7). The role of CXCL12 is to promote the processes of chemotaxis and migration^36^. For instance, in a murine model of arthritis, MSC expressing CXCL12 in an inflamed area of the bone suppressed the proliferation of osteoclasts and inflammatory stimuli^37^. Also senescent tumor cells produce CXCL12 to promote cancer cell migration and metastasis, and ultimately contributing to tumor development^38^. However, even though LA cells expressed higher levels of CXCL12, we did not observe any significant increase in the expression of matrix metalloproteinases (MMP) and other enzymes capable of degrading extracellular matrix, which are typical cellular signature of senescent tumor cells^39^. So, presumably, here MSC were not acquiring a tumorigenic phenotype, but rather reinforced their paracrine function, devoted to stimulate and support the fitness of the whole population.

It has been shown that the transplantation of senescent ear chondrocytes into the knee joint area of wild-type mice can exacerbate the effects of osteoarthritis, in comparison to transplantation of non-senescent chondrocytes^40^. On contrary, selective elimination of senescent cells potentially mitigates the effects of several age-dependent disorders^41^. For instance, the use of a potent senolytic drug—ABT263, inhibitor of the anti-apoptotic proteins BCL-2 and BCL-xL—successfully alleviated the ageing effects of total-body irradiation in mice bone marrow hematopoietic stem cells and muscle stem cells^42^. Similar to senolytic drugs, the idea of sorting MSC before transplantation in vivo would benefit the therapeutical outcomes, by reducing locally the inflammatory response in adjacent cells. We propose that targeting senescent cells is a promising approach for preventing or treating age-related diseases. Additionally, we did not observe deleterious effects on MSC by the sorting process via FACS—except a temporary slight increase in cell division time—which makes the protocol safe and reliable in terms of cellular health and future clinical applications.

As limitations, we have found that autofluorescence–based sorting had no significant improvements in the tri-lineage in vitro differentiation potentials of MSC (Fig. 4). Despite the reduction of SA-β-gal positive cells in the LA group, in comparison to control, the differentiation yield of MSC was almost identical, raising the hypothesis that cellular senescence and differentiation potential have mechanisms, which are not necessarily interconnected at all levels. Furthermore, in this study the results were affected by the inter-donor variability of the MSC populations. Starting cell population with different senescent levels resulted in high variance between data, which were normalized to the control. A population with a lower proportion of senescent cells will benefit less of the sorting because the removal of these cells will have a smaller impact on the overall cellular fitness of the population, in comparison to a population enriched with senescent cells.

In conclusion, we demonstrated the functionality of autofluorescence as a marker of MSC senescence. Sorting cell strategies to remove senescent cells or to delay senescence could be beneficial for both health span and life span of MSC. The method proposed here for extracting senescent cells from cultures is effective, fast and label-free. In perspective, MSC sorting based on autofluorescence is an economic assay which can be used on its own, or in combination with other tests, to control and improve cellular fitness and ultimately the outcomes of cell-based therapies.

## Materials and methods

All methods were performed in accordance with the relevant guidelines and regulations of this journal.

### MSC isolation and expansion

Bone marrow (BM) samples were harvested from the iliac crest of donors, as previously described^17^. The study was ethically approved by the ethics committee of canton Basel (Ethikkommission beider Basel [EKKB], Ref. 78/07). Written informed consent was obtained from all participants and this procedure was also accepted by the approving body. MSC were isolated from BM of three female donors (average age: 66 years; range: ± 11 years). The BM aspirates were immediately resuspended in 3.8% sodium citrate and phosphate buffered saline (PBS, AppliChem—Axonlab, Baden, Switzerland) and then filtered through a 100 µm cell strainer to remove clots (Falcon—Faust, Schaffhausen, Switzerland)^17^. Mononuclear cells were separated by H-Lympholyte Cell Separation Media gradient centrifugation (density 1.077 g/mL; Cedarlane—Bio Concept, Allschwil, Switzerland) in Leucosep tubes (Huberlab, Reinach, Switzerland) at 800 g for 15 min, washed with PBS, centrifuged again at 210 g for 10 min and plated at a density of 1 × 10^5^ cells/cm^2^ in tissue culture flasks (TPP—Faust) in α-MEM (Amimed—Bio Concept), supplemented with 10% foetal bovine serum (FBS, Gibco—LuBioScience, Lucerne, Switzerland), (100 units/mL) penicillin/(100 mg/mL) streptomycin (Pen-Strep, Gibco), 2.5 µg/ml amphotericin B (Sigma, Buchs, Switzerland) at 37 °C in a humid atmosphere containing 5% CO_2_. After two days, non-adherent cells were discarded, whereas adherent cells were cultured in growing medium consisting of DMEM/Ham’s F12 (Amimed), supplemented with 10% FBS, 1X Pen-Strep, 2.5 µg/ml amphotericin B and 5 ng/ml recombinant basic fibroblast growth factor (bFGF, Peprotech—LuBioScience), followed by media change three times per week. At 90% confluency, MSC were frozen and stored at -150ºC.

### MSC sorting

Frozen MSC at early passage number (ranging from P3 to P4) were thawed and cultured in growing medium before sorting. MSC were separated based on their autofluorescence (λ-ex 488 nm, λ-em 525) into three pools corresponding to the cells with the lowest (0–33%), middle (33–66%) and highest (66–100%) autofluorescence using FACS Aria instrument III (BD Biosciences, Fig. 1). As controls, MSC were either passed through the FACS without sorting them (unsorted group) or propagated in culture (control).

### MSC senescent phenotype characterization

After sorting, MSC were cultured in growing medium for three days and re-plated at the cell density of 1.25 × 10^3^ cells/cm^2^. Cell number and cell volume were measured with the Scepter cell counter (Millipore, Thermo Fisher Scientific, Zug, Switzerland).

Senescence-associated-β galactosidase (SA-β-gal) activity of MSC was determined using a chromogenic-based method, as described previously^18^. Cells were fixed with 2% formaldehyde and 0.2% glutaraldehyde in PBS (both AppliChem) followed by incubation over night at 37 °C in a freshly prepared staining solution at pH 6.0 consisting of: 5 mM K_3_Fe(CN)_6_, 5 mM K_4_Fe(CN)_6_, 2 mM MgCl_2_, 150 mM NaCl, 30 mM citric acid/phosphate buffer (all AppliChem) and 1 mg/mL 5-bromo-4chloro-3-indolyls β-D-galactopyranoside (X-Gal, Sigma)^17^. Following PBS washing, cells were counterstained with haematoxylin (Molecular probes) and SA-β-Gal positive cells were manually enumerated with bright field microscopy (Olympus, Volketswil, Switzerland) at × 10 magnification.

Autofluorescence data were acquired with CytoFLEX flow cytometer (Beckman Coulter Life Sciences, Nyon, Switzerland), using the excitation laser at 488 nm and detection optic at 525/50 nm. A 638 nm laser and 670/30 detector was used for live/dead stain TO-PRO-3 (Thermo Fisher Scientific). Resulting data files were analysed using FlowJo software v.10.0 software (Treestar, Ashland, OR, USA). To standardize cytometer settings between runs, 15 µm polypropylene calibration beads (PHCCBEADS, Millipore) were used and dead cells were excluded from analysis.

#### RNA isolation and RNA-seq

Total RNA was isolated from MSC (control, unsorted, low- and high-fluorescence) using QIAzol Lysis Reagent (Qiagen, Hombrechtikon, Switzerland) and Direct-zol RNA MiniPrep (Zymo, LucernaChem), according to the manufacturer’s instructions and kept at − 80 °C. Potential genomic DNA contaminations were removed by treating samples with DNase during RNA isolation protocol.

RNA-Seq assays were performed by Fasteris SA (Plan-les-Ouates, Switzerland). Poly-adenylated transcripts selection purified from 1 µg of total RNA for each sample was followed by cDNA libraries construction using the TruSeq Stranded mRNA Library Prep kit (Illumina, San Diego, CA, USA). Libraries underwent high-throughput sequencing in an Illumina NextSeq 500 sequencer and for each 100 million paired-end 50 bp reads were generated during the sequencing runs. Sequence data was subjected to quality control using an indexed PhiX reference sequence to estimate sequencing error. RNA-seq reads were mapped with TopHat (https://ccb.jhu.edu/software/tophat/index.shtml). Cufflink (https://cole-trapnell-lab.github.io/cufflinks/) was used to estimate transcript abundance, Cuffdiff for differential expression and CummeRbundfor (https://compbio.mit.edu/cummeRbund/) for data visualization. The threshold for significantly differentially expressed genes (DEG) was set with simultaneous false discovery rate (FDR) of < 0.05 and absolute value of log_2_ ratio ≥ 1.0.

Interaction networks were generated using STRING (www.string-db.org) database, version 11.0 (Search Tool for the Retrieval of Interacting Genes/Proteins)^43^. Based on the list of DEGs, a network was built and exported to Cytoscape 3.7.1 (www.cytoscape.org). The search for bottlenecks was performed with the CestiScaPe 2.2 plugin to identify the betweenness values of the nodes present in the networks^26^. Betweenness is a measure of how many times a node is part of the shortest path between all node pairs in the network, while node degree is the total number of connection of the single node.

GO Biological Processes were identified using the ToppGene website with DEG found to be differentially expressed between low- and high autofluorescence samples (https://toppgene.cchmc.org/enrichment.jsp).

#### Quantitative real-time PCR

Starting from 500 ng RNA per sample, cDNA was prepared using VILO cDNA Synthesis Kit (Invitrogen) in a reaction volume of 20 µL following kit instruction, and diluted 1:10 with ultrapure water. Real-time PCR reactions consisted in the primers listed in Supplementary Table S1 at a concentration of 250 nM, 5 µL cDNA template, and IQ SYBR Green Supermix (Bio Rad). Specific products were amplified by a quantitative PCR system (CFX96 Real Time System, Bio Rad). RT-PCR was carried out with the following settings: denaturation 95 °C—3 min (1 cycle), 95 °C—15 s, 60 °C—20 s and 72 °C—20 s (35 amplification cycles) in a final volume of 20 µL in 96-well plates (Bio Rad). Melting curve analysis was performed after each reaction. Relative gene expression was determined using the 2^−ΔΔCt^ method and the results were normalized to the expression of actin beta^44^ and peptidylprolyl isomerase A (PPIA)^17,45^.

#### MSC in vitro differentiation into osteogenic, chondrogenic and adipogenic lineages

MSC cultures were stimulated with the appropriate differentiation medium according to the conditions described below.

##### Chondrogenic differentiation

Collagen type I cubes (Biopad, Euroresearch, Italy) were used as scaffold to support cellular differentiation^17,46^. MSC (5 × 10^5^ cells) were seeded onto collagen cubes and kept for 30 min to allow adhesion, before addition of chondrogenic medium. MSC-collagen constructs were cultured for three weeks at 5% O_2_ in chondrogenic medium, consisting of: advanced DMEM + GlutaMAX (Gibco), 2.5% FBS, 100 units/mL penicillin, 100 mg/mL streptomycin, 2.5 µg/mL amphotericin B, 40 ng/mL dexamethasone (Sigma), 50 µg/mL ascorbic acid 2-phosphate (Sigma), 1 × Insulin-Transferrin-Selenium X (Gibco), and 10 ng/ml transforming growth factor-β1 (TGF-β1, Peprotech).

Glycosaminoglycan (GAG) accumulation was determined as chondrogenic marker. GAG accumulation was quantified with alcian blue binding assay after six hours digestion of three cell-constructs per sample at 60 °C with 125 µg/ml papain (Sigma) in 5 mM L-cysteine-HCl (Fluka), 5 mM Na-citrate, 150 mM NaCl and 5 mM EDTA (all AppliChem). GAG accumulation was determined by binding to alcian blue (Fluka, Sigma) and quantified using chondroitin sulphate (Sigma) reference standards^47^.

##### Osteogenic differentiation

MSCs in monolayer at a density of 5 × 10^3^ cells/cm^2^ were differentiated in STEMPRO Osteogenesis Differentiation Kit (Gibco) for three weeks^17^. Calcium content was determined using the Calcium CPC LiquiColor test kit (StanBio, Schwetzingen, Germany). Cells were washed with PBS, incubated with 0.5 N HCl for 30 min at room temperature and then with O-Cresolphthalein complexone in alkaline solution. Calcium concentration was measured (absorbance at 405 nm) and quantified with standards.

##### Adipogenic differentiation

MSC were cultured in monolayers at a density of 2.5 × 10^4^ cells/cm^2^, alternating two different culture conditions^17^: adipogenesis maintenance medium—DMEM/Ham’s F12 + GlutaMAX, 2.5% FBS, 1X Pen Strep, 2.5 µg/mL amphotericin B and 170 mM insulin; and adipogenesis inducing medium—adipogenesis maintenance medium supplemented with 1 µM dexamethasone, 0.5 mM 3-Isobutyl-1-methylxanthine and 0.5 mM indomethacin (all Sigma). After two weeks, lipid droplets were stained with Oil Red O (Sigma), and the dye content was quantified after isopropanol elution and spectrophotometry by measuring the absorbance at 492 nm.

#### Immunoblot analyses

Total protein was isolated from MSC before and after sorting, and processed as previously described^17^. Briefly, cells were lysed in 150 mM NaCl, 1% Triton X-100, 0.5% Na deoxicholate, 0.1% SDS, 50 mM Tris, pH 8.0, (all AppliChem) and 1X protease inhibitor cocktail (Sigma) for 30 min, centrifuged at 800 g for 10 min (both at 4 °C) and protein extracts (10 µg) were fractionated by Mini-Protean TGX 4–15% gradient polyacrylamide gels, and semi-dry blotted to a nitrocellulose membrane (both Bio Rad). The nitrocellulose membranes were incubated with mouse monoclonal antibodies against anti-p53 (mouse, OP43, Calbiochem, Sigma), anti-p21 (rabbit, 2947, Cell Signaling Technology—Bio Concept), anti-p16 (mouse, NA29, Calbiochem), anti-RB (mouse, OP66, Calbiochem) and housekeeping gene β-actin (mouse, HRP conjugate, 12,262, Cell Signaling Technology). Blocked membranes were probed overnight at 4 °C with primary antibodies diluted in 1% milk (Rapilait, Migros, Switzerland) in PBS, followed by HRP-conjugated mouse or rabbit secondary antibody (both LuBioScience) in 1% milk in PBS, for one hour at room temperature. Membranes were developed with LumiGlo Reserve Chemiluminescent Substrate Kit (LumiGlo Reserve KPL—Bio Concept). Image acquisition was performed with a digital SLR camera (Nikon D600, Nikon, Zürich, Switzerland) and the results were normalized to the relative amount of β-actin with ImageJ (version 1.46r, ImageJ inc.).

#### Statistical analysis

Since n = 3 in our experimental layout, we plotted the data as box plots—generated with SPSS for Windows (version 24.0, SPSS Inc.)—for a better interpretation of results, without implementing any statistical inferences and possibly misleading *p* values^48^. Gene expression validation of RNA-Seq results is represented as clustergram, created using CFX Manager (version 3.0, Bio Rad).

## Supplementary information

Supplementary Information

## Data Availability

The datasets generated during the current study are available from the corresponding author on reasonable request. The Fastq files generated will be deposited in the Gene Expression Omnibus (GEO) database.
