# Supplementary material for: Autofluorescence-based sorting removes senescent cells from mesenchymal stromal cell cultures
Source: Sci Rep. 2020 Nov 5;10:19084. doi: 10.1038/s41598-020-76202-2 (PMC7645702; doi:10.1038/s41598-020-76202-2)
Supplement: Supplementary file 1 — Supplementary Information [file 41598_2020_76202_MOESM1_ESM.docx]

**Autofluorescence-based sorting removes senescent cells from mesenchymal stromal cell cultures**

by Alessandro Bertolo^1^, Julien Guerrero^2^, Jivko Stoyanov^1*^

^1^ Swiss Paraplegic Research, Nottwil, 6207, Switzerland.

^2^ Tissue Engineering for Orthopaedics & Mechanobiology (TOM), Department for Biomedical Research (DBMR), University of Bern, Bern, 3008, Switzerland.

* jivko.stoyanov@paraplegie.ch

**Figure S1** | **Comparative analysis of RNA-seq data acquired from MSC sorted by autofluorescence.** MSC were divided in control and unsorted groups, and low (LA) and high (HA) autofluorescence groups. Data was plotted by group for all genes for count versus dispersion **(a)**, principal component analysis (PCA) and multi-dimensional scaling (MDS) plots for gene-level features **(b)**.


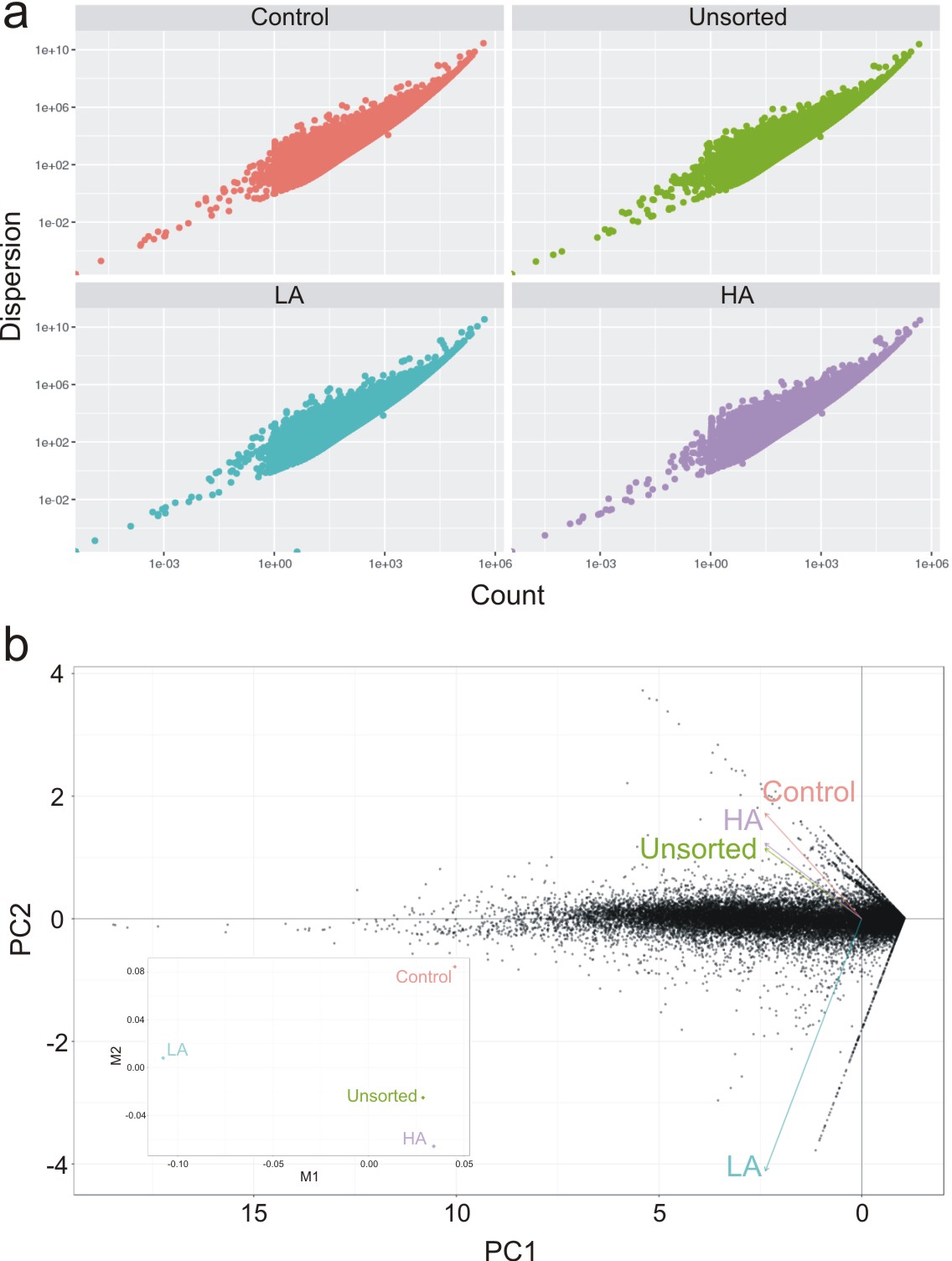


**Figure S2** | **Full-length immunoblots at different exposure times.** Prior to antibody incubations, membranes were cut according to the size of the protein of interest. Samples were loaded in the following order, starting from the left side: Control, Unsorted, LA, MA and HA.


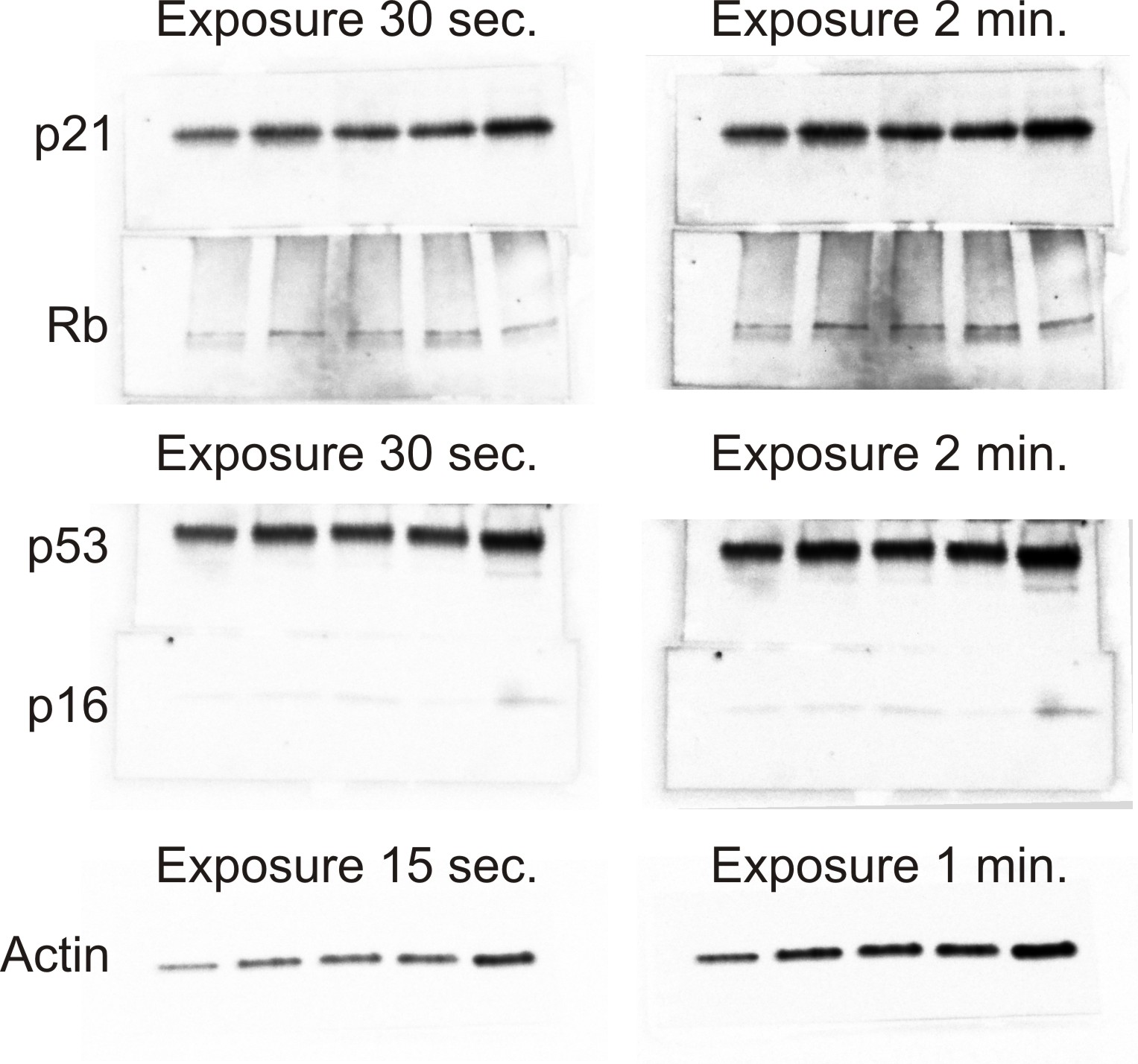


**Table S1.** Primer pairs of human genes used in quantitative RT-PCR (F = Forward, R = Reverse. bp = base pairs)

| **Gene** | **Primer nucleotide sequence (5’ to 3’)** | **Amplicon** |
| --- | --- | --- |
| (NCBI Reference Sequence) | | **(bp)** |
| **Housekeeping genes** | |  |
| **PPIA ^53^** | F - GTCAACCCCACCGTGTTCTT | 97 |
| (NM_021130) | R - CTGCTGTCTTTGGGACCTTGT |  |
| **Actin beta** | F - CCAACCGCGAGAAGATGA | 97 |
| (NM_001101.5) | R - CCAGAGGCGTACAGGGATAG |  |
| **Senescence associated genes** | |  |
| **p16^INK4a^** | F - GTGGACCTGGCTGAGGAG | 132 |
| (NM_000077) | R - CTTTCAATCGGGGATGTCTG |  |
| **p18^INK4C^** | F - AGAGATCTGTAGCGTAGGTACGTG | 74 |
| (NM_078626) | R - ACATACATTCCTGGTTAATGACTCC |  |
| **p21^CIP1^** | F - CGAAGTCAGTTCCTTGTGGAG | 111 |
| (NM_000389) | R - CATGGGTTCTGACGGACAT |  |
| **E2F1** | F - TCCAAGAACCACATCCAGTG | 75 |
| (NM_005225) | R - CTGGGTCAACCCCTCAAG |  |
| **CDCA7 ^32^** | F - CTGCCCAGAAGCCGTCGCTC | 222 |
| (NM_031942) | R - GAACTGGCCTCGAACGCCCC |  |
| **ANKRD1 ^32^** | F - CGGAACCTGTGGATGTGCCTACG | 245 |
| (NM_014391) | R - TCCTCCACGGCTTGCCCAGT |  |

**Table S1.** Human genes used in quantitative RT-PCR. (Continuation)

| **Gene** | **Primer nucleotide sequence (5’ to 3’)** | **Amplicon** |
| --- | --- | --- |
| (NCBI Reference Sequence) | | **(bp)** |
| **RNA-Seq validation** | |  |
| **BEX1** | F - CCCTGCTTTCGAATTTACATGTTCA | 107 |
| (NM_018476.4) | R - AACACAATATTCAATTAGAAGCTGG |  |
| **C1R** | F - AAGATTCCTCGGTGCTTGC | 82 |
| (NM_001354346.2) | R - CTTTTTGCCCTCCGATGAT |  |
| **C7** | F - GAAAGCACACAATGCGAAGA | 62 |
| (NM_000587.4) | R - AAGCAATGTGGTTCAAGCAAC |  |
| **TPD52L1** | F - CTGTCACAAGCCTCAAGACG | 111 |
| (NM_003287.4) | R - AGCCTCCTGCCAAGCTCT |  |
| **VCAM** | F - TGCACAGTGACTTGTGGACAT | 92 |
| (NM_001078.4) | R - CCACTCATCTCGATTTCTGGA |  |
| **POLG** | F - GGGCACAATGTTTCCTTTGA | 94 |
| (NM_002693.2) | R - TGTGCATGCTCATGGTGTC |  |
| **SERPINB2** | F - ACCTCATACAGATTCTGCAAAGATT | 69 |
| (NM_001143818.1) | R - GCCACACAAAGATCCTCCAT |  |
| **PLAT** | F - GAAGAGAGGGCTCTGCTGTG | 114 |
| (NM_033011.4) | R - TCGCTGCAACCTTGGTAAG |  |
| **MYCT1** | F - GGCCTCAACAGAACTGGATT | 61 |
| (NM_025107.2) | R - GGCTGAGGTTGCTTCGAC |  |
| **KRT34** | F - GTGTGAGATCAACACGTACCG | 93 |
| (NM_021013.3) | R - GAGTTGCCACTAGCATTGGTG |  |
| **TMEM171** | F - GATCCAGATTATGGAGCCTGTC | 98 |
| (NM_173490.8) | R - CAGAAGCTGAAGATTCAGGAAAG |  |
| **ESM1** | F - CATGGATGGCATGAAGTGTG | 106 |
| (NM_007036.5) | R - GGTGCCGTAGGGACAGTCT |  |

**Table S1.** Human genes used in quantitative RT-PCR. (Continuation)

| **Gene** | **Primer nucleotide sequence (5’ to 3’)** | **Amplicon** |
| --- | --- | --- |
| (NCBI Reference Sequence) | | **(bp)** |
| **CAV1** | F - GAGCTGAGCGAGAAGCAAGT | 130 |
| (NM_001753.5) | R - TCCCTTCTGGTTCTGCAATC |  |
| **EPGN** | F - TGCATCAACGGTGCTTGT | 78 |
| (NM_001270989.2) | R - TTCTCCAGTATAACCAGTAAAACACCT |  |
| **CXCL12** | F - TTGACCCGAAGCTAAAGTGG | 73 |
| (NM_000609.7) | R - CCCTCTCACATCTTGAACCTCT |  |
| **FGFR2** | F - CCTGCGGAGACAGGTAACAG | 82 |
| (NM_000141) | R - GCGTGTTGTTATCCTCACCA |  |
| **FMO2** | F - TGTGTGGATGAGGGACTTGA | 102 |
| (NM_001460.5) | R - GATACTTGCTCGGCCATCTT |  |
| **NDNF** | F - CTTCTCACCAAAAAGTCACCTTC | 75 |
| (NM_024574.4) | R - TCCCATCTCTTCTCACTTGGAT |  |
